# Associations between sociodemographic factors, health spending, disease burden, and life expectancy of older adults (70 + years old) in 22 countries in the Western Pacific Region, 1995–2019: estimates from the Global Burden of Disease (GBD) Study 2019

**DOI:** 10.1007/s11357-021-00494-z

**Published:** 2022-01-08

**Authors:** Alex Molassiotis, Stephen W. H. Kwok, Angela Y. M. Leung, Stefanos Tyrovolas

**Affiliations:** 1 School of Nursing, The Hong Kong Polytechnic University, Hung Hom, Kowloon, Hong Kong SAR; 2 Parc Sanitari Sant Joan de Déu, Fundació Sant Joan de Déu, CIBERSAM, Barcelona, Spain

**Keywords:** Aging, Older adults, Western Pacific, Non-communicable diseases, Life expectancy, Disease burden, Sociodemographic factors

## Abstract

The needs of the aging populations are putting increasing burden on healthcare particularly in the Western Pacific Region (WPR), which is the home of aging economies such as Hong Kong and Japan alongside rapid increases in older people in low- and middle-income countries (LMICs). However, little is known about the associations between sociodemographic factors, disease burden, and life expectancy in WPR. The current study conducted secondary analysis on the Global Burden of Disease (GBD) estimates of populations aged 70 years and older in WPR countries between 1995 and 2019. Correlation tests, linear mixed regressions and generalized additive mixture models were run to examine the associations of interest. Unsupervised machine learning was conducted to segment the data automatically, at cluster analysis. The sociodemographic development index was found an important factor to the disease burden in terms of Years Lived with Disability (YLD) rate and mortality rate among older adults. YLD rate and mortality rate of non-communicable diseases (NCDs) attributable to different risk groups were significant contributors to higher expectation of Lost Healthy Years (LHE) and shorter life expectancy respectively in the context of rising YLD rate, particularly in LMICs. Three clusters of countries with similar characteristics were identified. NCDs were the most significant contributors to shorter life expectancy within which the time living in poor health did not improve. Better management of NCDs in aging populations should be emphasized in all countries. The country clusters may help in a more regional strategic planning.

**Supplementary Information:**

The online version contains supplementary material available at 10.1007/s11357-021-00494-z.

## Introduction

The global population is generally aging in the last 30 years. Life expectancy at birth rose from around 67 years in 2000 to 73.5 years in 2019 [1]. However, ageing is followed by age-associated diseases and the healthcare needs and costs incurred have likely been an increasing burden in this population. In the Western Pacific Region, ageing-related epidemiological data allowing healthcare planning is scarce but the healthcare needs could be increasingly high. There is a significant demographic shift in the Western Pacific region in which those over the age of 60 years old outnumber those less than 5 years; the ageing index is among the highest in the low and middle income countries (LMICs) in the region, and in many of the countries of the region the population of older people will double by 2030 [2]. For instance, Japan has the second longest life expectancy in the world after Hong Kong SAR, China, and the longest life expectancy among OECD countries [3, 4] with a rapidly increasing older population. However, the transition to an ageing population in LMICs is much faster than that of the higher income countries in the region. Developed economies have higher sociodemographic index (SDI), a measure of a country’s level of development, than developing nations [5]. The SDI was found to be positively associated with life expectancy at the global level [1]. However, data from the Western Pacific region, with its particular ageing society characteristics, diverse cultures and healthcare systems, the size of its population (particularly rural population) and with the majority of the countries being LMICs, will be useful for regional planning.

Given an estimated life expectancy, a key research interest is the proportion of it that would be lived in good health. The GBD 2019 Demographics Collaborators [1] found that both the healthy life expectancy (HALE) and the equivalent years of healthy life lost increased along with SDI or time. The LHE (expectation of Lost Healthy Years) is the product of life expectancy and LHE fraction, which is estimated from Years Lived with Disability (YLD) per capita [6, 7]. YLDs from NCDs and injuries have been increasingly contributing to burden of diseases since 1990 [7]. The findings indicated that NCDs is becoming prevalent in both developed and developing countries.

Disability Adjusted Life Years (DALYs) is a unit of disease burden in GBD studies. It is the sum of the YLD rate and the product of mortality rate and standard life expectancy [7, 8]. Overall, the annualised rate of change in DALY rate fell when SDI increased [7]. However, improvements in age-standardised DALY rates have begun to slow down or reverse in high SDI economies [7]. NCDs remained to be significant contributors to disease burden. For instance, among those aged 50 or above in 2019, stroke and ischaemic heart disease were the top-ranked causes of DALYs [7], and high systolic blood pressure was the leading risk factor for attributable DALYs [9].

In the period between 1995 and 2016, the global health-spending rose by 4–6%, and the governments increasingly made the largest contributions [10, 11]. In the Western Pacific Region, the Japanese achieved universal health coverage early in the 1960s [12]. The healthcare providers were reimbursed by the government through public insurance based on a nationally uniform fee which was set and revised by the government [13]. At the same time, the co-payment rate was as low as around 14% [2], which was comparable to the 18% in Australia [14]. On the other hand, some developing countries have begun increasing their coverage rates. In the Philippines, the coverage rate rose from 70 to 90% between 2010 and 2016 [15]. In China, the coverage rate climbed from around 30% in early 2000s to over 90% in 2010 [16]. Moreover, the Chinese government started implementing time-of-service co-payment programmes in one hundred cities since 2014 [17].

By implementing the policy of universal health coverage, the health-related financial burden on governments might inevitably grow alongside the rising disease burden associated with ageing populations. In other words, the increase in disease burden could be a factor to additional health spending which needs to be controlled. Zhai et al. [18] projected the Chinese data and showed that spending on NCDs would rise to at least three fourths of the total health spending by 2035, and the expenditure might be reduced if the prevalence rates of risk factors such as smoking and hypertension could be lowered.

Nevertheless, given that countries with higher SDI would have higher income and lower disease burden than economies with lower SDI, the associations between health spending and YLD rates as well as mortality rates, which are determinants of life expectancy and disease burden, require further study. Moreover, the relative strength and significance of associations between the YLD rate and mortality rate of level 1 risk-cause pairs and the LHE and life expectancy respectively need to be investigated. Data with respect to locations and sexes in addition to other features such as sociodemographic factors, health spending, disease burden, and life expectancy at older ages might be segmented into clusters to which the countries belong, which may be important for regional planning. The current data on ageing issues in the Western Pacific Region, with the exception of high-income countries, is scarce. This is probably a result of that a majority of the countries in this region were developing ones which had to deal with other health priorities, had shorter life expectancy than developed countries, and lacked an urgency in collecting such data as in the western industrial world. Hence, the aim of the current study was to investigate the proposed associations between these features; as well as to identify clusters of countries for generalizable health planning and interventions in the Western Pacific Region.

Study objectives

1. To assess life expectancy and healthy life expectancy in those 70 years old and above in Western Pacific region countries.
2. To identify clusters of Western Pacific countries with similar ageing-related characteristics and health risks that would allow regional health policy initiatives rather than country-based ones.
3. To compare the differences in sociodemographic factors, disease burden, and life expectancy between clusters of Western Pacific countries in those over 70 years.
4. To analyse sociodemographic factors and healthcare spending associated with mortality and YLDs attributable to epidemiological risk factors in those over 70 years.
5. To identify the strength of associations between mortality and YLDs and life expectancy in the Western Pacific population over 70 years old.

## Methods

### GBD measures, cause list, and Western Pacific Region

We analysed results of the GBD 2019 study (which includes data from 1995 to 2019) to evaluate Western Pacific trends in epidemiological patterns and disease burden as well as their relationship with sociodemographic factors and health spending for the population aged above 70 years. The analyses employed established GBD 2019 summary measures of morbidity and mortality, including death rates, YLDs, LE, HALE and LHE at 70 + years old. Seventy years and older is the GBD cut-off used to define older age. This threshold was selected based on availability of age groups in the GBD study (reflecting only the population over 65 years old). Additional details of methods used to estimate mortality rates, YLDs, including all other analytic approaches for the assessment of relative morbidity and mortality from individual diseases and injuries, are available in the latest GBD publications [1, 7, 9]. GBD data are publicly available and can be downloaded at http://ghdx.healthdata.org/gbd-results-tool. In the GBD 2019 study, causes of mortality and morbidity are defined using a four-level categorisation to deliver mutually exclusive and collectively exhaustive levels. The GBD 2019 study estimates the impact of 369 diseases and injuries as well as 87 risk factors between 1990 and 2019. The GBD study is organised by a geographical categorisation of super-regions and regions, with 204 countries and territories assigned within these areas. Within this topographical categorisation, the GBD study estimates seven fatal and non-fatal causes at Level 1, 22 at Level 2, 169 at Level 3, and 297 at Level 4. The full GBD cause hierarchy, including corresponding International Classification of Diseases (ICD)-9 and ICD-10 codes, is published in detail in previous GBD publications [19, 20]. For the current study only 22 Western Pacific countries were included in the analysis. These were countries in the East Asia (China, Mongolia, Japan, and South Korea); countries in the Southeast Asia (Cambodia, Laos, Vietnam, the Philippines, Malaysia, Brunei, and Singapore); and countries in the Oceania (Australia, New Zealand, Papua New Guinea, Fiji, Kiribati, Marshall Islands, the Federated States of Micronesia, Samoa, Solomon Islands, Tonga, and Vanuatu).

### Mortality, life expectancy, and healthy life expectancy estimates

GBD mortality is estimated using standardized modelling processes—most commonly, the Cause of Death Ensemble model, which uses covariate selection and out-of-sample validity analyses and generates estimates for each location-year, age group, and sex. Additional details, including model specifications and data availability for each cause-specific model, can be found in the GBD 2019 mortality and causes of death publications [1]. In the current study we used the mortality rates for those 70+ years old covering information for the period 1995 to 2019. For the calculation of LE, the GBD is using a specific model life table system (which identifies a reference table of each location, year, and sex, based on the nearest matches in the GBD empirical life table database) [1]. Using the GBD 2019 dataset in the current study, mean life expectancy (LE at 70+) was used by our team, as the average expected years of life remaining at age 70-94 years from 1995 to 2019. GBD also uses HALE to estimate the number of years that people can expect to live in good health [21]. In a similar way as abovementioned for LE at 70+ we estimated the HALE at 70+ using the age groups 70-94 from 1995 to 2019. Expectation  of Lost Healthy Years (LHE, =LE-HALE) at 70 + is the expected years of life living in poor health for those between 70–94 years old, and the fraction of LHE at 70 + is the proportion of LHE divided by LE at 70 + [6].

### Years lived with disability and risk factors

GBD study uses epidemiological data from systematic literature reviews, health surveys, surveillance systems, disease registries, and hospital and claims databases to generate cause-specific and sequela-specific prevalence and incidence estimates. GDB generated these estimates using a variety of modelling approaches, of which Bayesian meta-regression compartmental modelling in DisMod-MR 2.1 was the most common [22]. GBD then used a microsimulation framework to adjust for comorbidities and calculated YLDs for each cause by multiplying prevalence and corresponding disability weights for each sequela of each cause [23, 24]. In this research work we used the YLDs rates focused specifically for the population 70+ years old [7] from 1995 to 2019. The GBD 2019 comparative risk assessment framework classified each of 87 risk factors and clusters of risk factors into one of three categories: behavioral, environmental/occupational, or metabolic. Data on risk factor exposure levels were identified, evaluated, and modelled in the GBD study using similar approaches to nonfatal models. Quantitative relative risk was estimated for each risk-outcome pair, and population-attributable fraction statistics were calculated using standard GBD CRA methods [9]. We used these risk outcome paired estimates for the population 70+ and for the years 1995-2019. 

### Socioeconomic, healthcare access and quality indicators, health spending and other variables

To further assess the effects of socioeconomic factors, health system performance on Western Pacific health metrics, we employed the Socio-demographic Index (SDI), the Healthcare Access and Quality (HAQ) Index and healthcare spending estimates respectively. Also, other variables such as years of education per capita and the population fertility rate were used. The mean years of education per capita was the average years of schooling and single-year distribution of educational attainment annually by sex for adults ages 25–29 for 1995 to 2019 [25]. The fertility rate was the annual estimates for total fertility under 25 years (TFU25) per 1000 women for 1995 to 2019 [26]. SDI is a composite indicator based upon the country-level income per capita, the average educational attainment among individuals over age 15, and the total fertility rate among women under 25. The SDI ranges from 0 to 1 [7]. Each country’s income level (high (HICs)-, middle (MICs)-, low (LICs)-) was based on the World Bank’s classification [27]. HAQ Index is a composite metric developed following GBD 2016 that is based on comparative mortality rates for health-care-sensitive diseases, standardized to risk exposure level, and is meant to quantify the overall performance of health systems. The HAQ Index ranges from 0 to 100 [28]. The measures of healthcare spending consist of total healthcare spending (THS) per capita, THS per GDP, government health spending per THS, prepaid private spending per THS and out-of-pocket (OOP) spending per THS for 1995 to 2019 [29, 30].

### Data analysis

**Cluster analysis**: Two-step cluster analysis was used to explore the number and characteristics of clusters in the time series data from 1995 to 2019 with respect to Western Pacific Region countries. The categorical variables were country and sex. The continuous variables were GDP per capita, education years, fertility rate, THS per capita, THS per GDP, government spending per THS, prepaid private spending per THS, OOP spending per THS, HAQI frontier, YLD rate, mortality rate, HALE, and LHE fraction at 70+ years old. A pre-cluster step was used a sequential clustering approach, which followed by the procedure of constructing a modified cluster feature (CF) tree. More information on the cluster analysis can be found in the appendix. **Correlation analysis: **Associations among several factors such as the sociodemographic factors, health spending, disease burden and life expectancy at 70+ were tested with Spearman’s rank-order correlation tests. Spearman’s correlation was tested on the pooled dataset as well as for years 1995 and 2019. All P-values are based on two-sided tests. A P-value <0.05 was considered as significant. **Longitudinal Regression analysis: **Analysis was based on time-series from 1995 to 2019, in order to ensure the maximum data availability. Linear mixed effects (LME) models and generalized additive mixture (GAM) models were built to estimate the parameters of YLD rate and mortality rate, LHE, and life expectancy at 70 + with respect to several characteristics (more detail on the models are presented on the supplementary appendix 1).  The software used for correlation tests and cluster analysis was IBM SPSS 25 (Armonk, NY: IBM Corp.). R 4.1.0 (Vienna, Austria: R Foundation) on RStudio 1.4.1717 (Boston, Massachusetts: RStudio) was used to run LME and GAM models.

## Results

### Life expectancy and healthy life expectancy at the age of 70 years

Table 1 shows LE and HALE at age 70 years. The highest LE in males was observed in Singapore, followed by Japan, Australia, New Zealand and Korea but HALE was 3.9–4.64 years less than LE. In females the picture was similar, although China, Malaysia, Vietnam, Cook Islands and Tonga followed with relatively high LE. HALE was lower by 5 + years in the high income countries for females with the same pattern across all countries, indicating that while females have longer LE at 70 years old, the years living in poor health also increase in these countries.

**Table 1:** Life expectancy (LE) and healthy life expectancy (HALE) in Western Pacific countries at the age of 70 years old, for 2019

Country	LE (Male)	HALE (Male)	LE (Female)	HALE (Female)
China	11.82	8.87	15.13	10.59
Cambodia	10.2	7.31	12.18	8.58
Laos	10.52	7.71	12.12	8.67
Malaysia	12.18	8.79	13.26	9.35
Philippines	11.53	8.35	13.46	9.54
Vietnam	10.73	7.83	14.34	10.28
Fiji	9.39	6.58	11.51	7.93
Kiribati	8.32	5.99	9.15	6.54
Marshall Islands	10.13	7.28	10.06	6.97
Micronesia	9.19	6.75	9.77	6.92
Papua New Guinea	10.05	7.31	11.4	7.87
Samoa	11.44	8.33	11.9	8.31
Solomon Islands	8.13	6.01	8.32	5.97
Tonga	11.01	8.12	14.4	10.14
Vanuatu	9.76	7.18	10.54	7.42
Mongolia	9.31	7.14	12.11	9.22
Brunei	9.72	7.01	12.76	9.1
Japan	16.47	12.33	20.41	14.86
Korea	15.13	11.23	18.32	13.34
Singapore	16.74	12.7	19.45	14.5
Australia	15.96	11.32	18.32	12.75
New Zealand	15.47	11.05	17.56	12.31
Cook Islands	12.57	8.88	14.74	9.99
Nauru	8.95	6.51	9.8	6.84
Niue	10.46	7.43	12.71	8.63
Palau	10.25	7.3	11.33	7.75
Tuvalu	10.78	7.9	10.93	7.63

Data on this table are reflecting LE and HALE for the population at the age of 70 (age group 70-74), for 2019. LE and HALE information on the rest of the age groups for the period 1995 to 2019 can be found at http://ghdx.healthdata.org/gbdresults-tool

## Clustering of countries and YLD and mortality country profiles

In the exploratory cluster analysis, three clusters of countries were identified (Table 2) based on 15 input variables (Supplementary Fig. 1). The total health spending (THS) per capita in PPP and out-of-pocket spending per THS were the two most important inputs defining the clusters. THS per capita in cluster 1 (developed countries) was around eight and ten times of the THS in cluster 2 and 3 respectively. On the other hand, nearly half of the THS was contributed by out-of-pocket payment in cluster 3, while the OOP spending contributed one fourth of the THS in cluster 1. In cluster 2, the percentage of THS attributed to OOP expenditure was only 14%, much lower than that both the other clusters.

**Table 2:** Centroids of clusters with respect to continuous inputs and cluster membership of countries in populations older than 70 years old in the Western Pacific Region, 1995–2019

	Cluster 1	Cluster 2	Cluster 3	Combined	Importance
	*n* = 250	*n* = 544	*n* = 300	*N* = 1094	
	Mean (SD)	Mean (SD)	Mean (SD)	Mean (SD)	
Total health spending per capita in PPP	3076 (1135·43)	407·36 (585·47)	319·66 (278·28)	993·15 (1331·44)	0·9978
Out-of-pocket spending per THS	0·26 (0·13)	0·14 (0·07)	0·48 (0·09)	0·26 (0·17)	0·9409
Healthy life expectancy, age 70 +	6·82 (0·97)	4·25 (0·63)	5 (0·52)	5·04 (1·23)	0·8794
Deaths per 100K, all risk-cause, age 70 +	2873·65 (820·81)	6733·56 (1374·28)	5340·61 (1192·66)	5469·52 (1954·99)	0·7301
HAQI frontier	87·99 (2·63)	67·96 (10·25)	66·8 (9·47)	72·22 (12·34)	0·512
Mean years of education, age 25–29	13·56 (1·01)	8·86 (2·41)	8·24 (2·42)	9·76 (3·01)	0·5023
Fertility per 1000, age 10–24	0·27 (0·15)	1·04 (0·37)	0·93 (0·4)	0·84 (0·46)	0·4726
Government spending per THS	0·64 (0·16)	0·67 (0·15)	0·37 (0·12)	0·58 (0·19)	0·4529
Prepaid private spending per THS	0·1 (0·05)	0·02 (0·03)	0·09 (0·05)	0·06 (0·06)	0·4061
YLDs per 100K, all risk-cause, age 70 +	8582·29 (1164·86)	11,641·51 (1834·1)	10,723·56 (934·75)	10,690·7 (1919·99)	0·3907
GDP per capita, PPP (current international $)	38,233·32 (18,347·31)	10,214·29 (19,907·8)	6908·81 (6488·84)	15,710·74 (20,914·68)	0·3296
Total health spending per GDP	0·07 (0·02)	0·06 (0·04)	0·04 (0·01)	0·06 (0·03)	0·0735
LHE fraction, 70+ years old	0·3 (0·02)	0·31 (0·02)	0·31 (0·02)	0·31 (0·02)	0·0032
Countries	Japan Republic of Korea Singapore Australia New Zealand	Fiji Kiribati Marshall Islands Micronesia (Federated States of) Papua New Guinea Samoa Solomon Islands Tonga Vanuatu Mongolia Brunei Darussalam	China Cambodia Lao People's Democratic Republic Malaysia Philippines Viet Nam		

Cluster analysis applied on 1995-2019 GBD data. Each location, by sex, by year, was considered a case. Silhouette measure of cohesion and separation = 0·4. *THS* total health spending. *GDP* gross domestic product. *PPP* purchasing power parity. *HAQI frontier* healthcare access and quality index computed based on relationship between HAQI and SDI. *HALE* healthy life expectancy. *YLD* years lived with disability. *LHE* equivalent years of healthy life lost

The importance of government spending, prepaid private spending and GDP per person were lower than that of OOP spending in the clustering. The THS contributed by the government was less important in the clustering, but the percentages were significant. Over two thirds of the THS in cluster 1 and 2 were paid by governments, while more than one-third of the THS was sponsored by the governments in cluster 3. The contributions from prepaid private spending were much smaller, which were less than 10% of the THS, particularly in cluster 2. Though the GDP per capita in cluster 1 was much higher than the value in the other two clusters, the THS per GDP between clusters was similar ranging between 4 and 7%.

Furthermore, HALE at 70 + was also an important input in the clustering. The mean HALE was 7, 4, and 5 years in cluster 1, 2, and 3 respectively. Nonetheless, the LHE fractions at 70 + were similar across clusters, which were nearly 30%. The mortality rate, fertility rate, as well as YLD rate followed the reverse order where the rates in cluster 1 were the lowest and the rates in cluster 2 were the highest. Healthcare access and quality (HAQI) and years of education had moderately high level of importance to the clustering. The mean HAQI frontier in cluster 1 was 88, which was higher than the 67 in the other two clusters. Furthermore, the years of education per capita in cluster 1 was the longest, while the education years in the other two clusters were shorter.

**Fig. 1:**
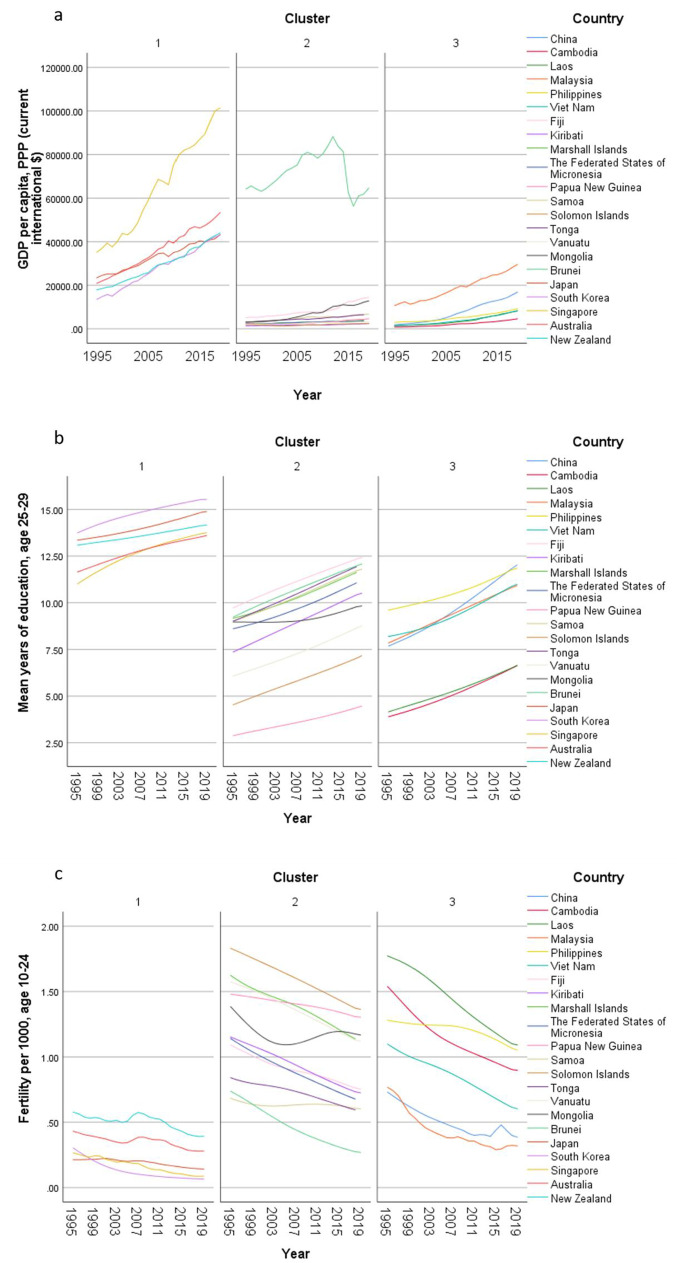

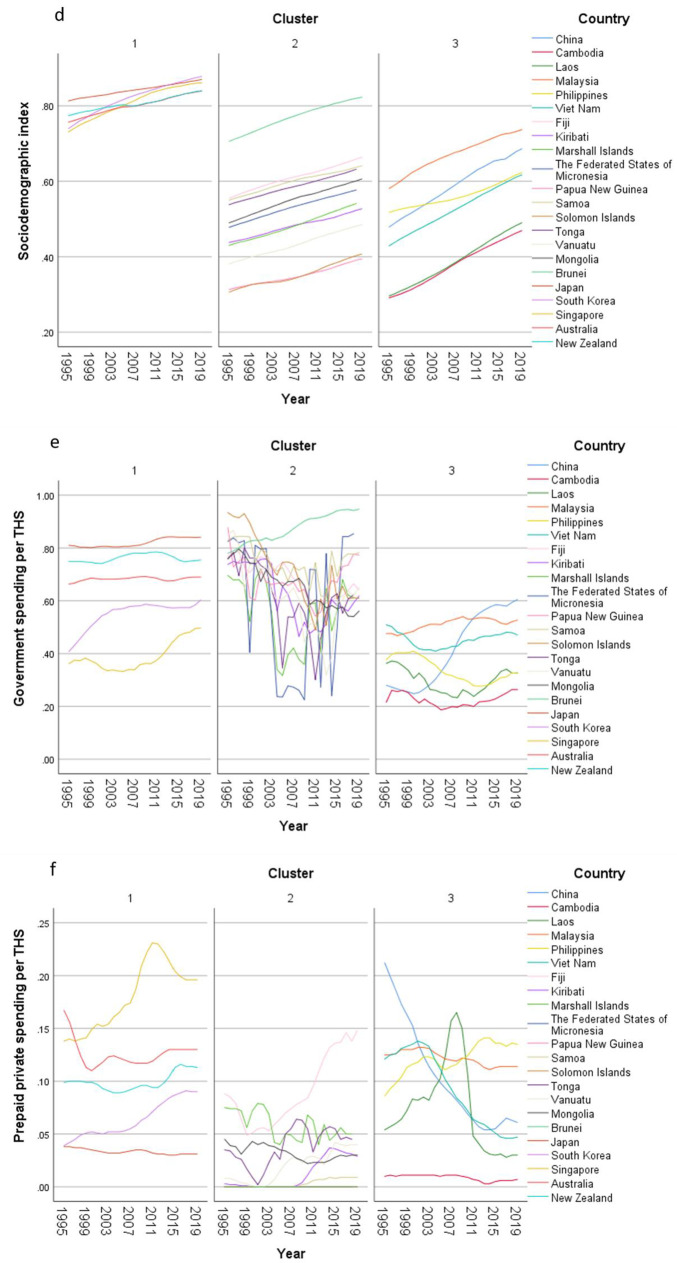

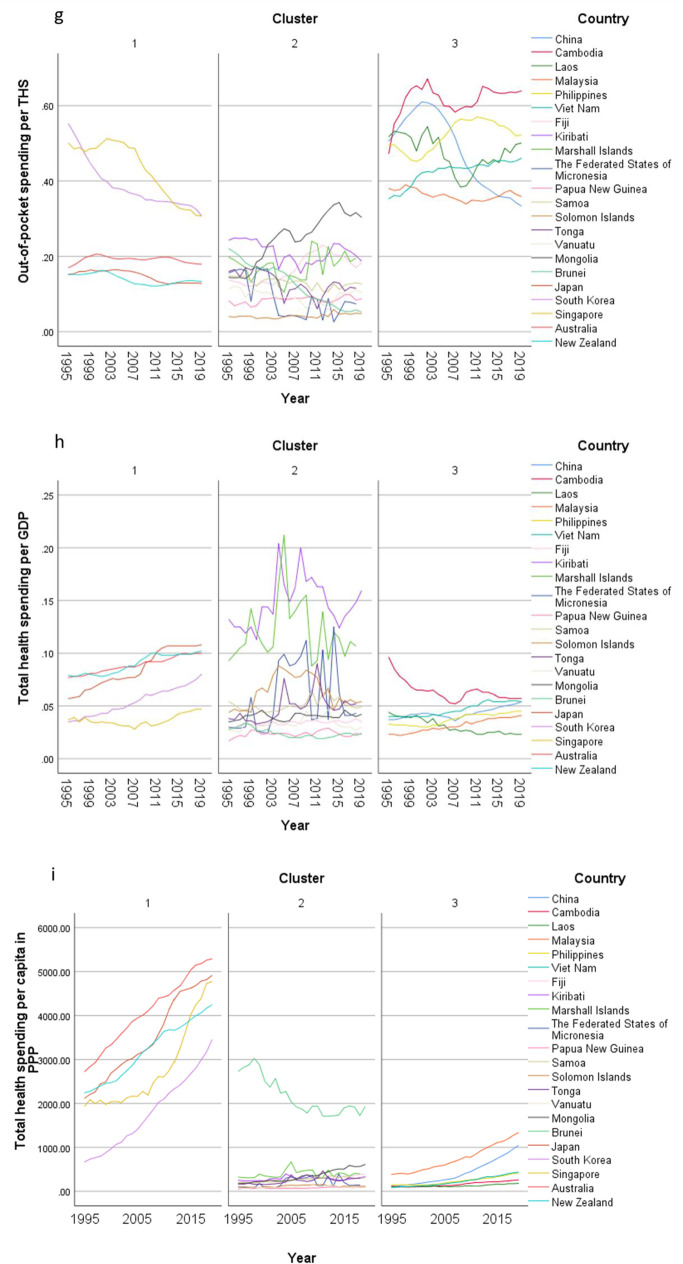

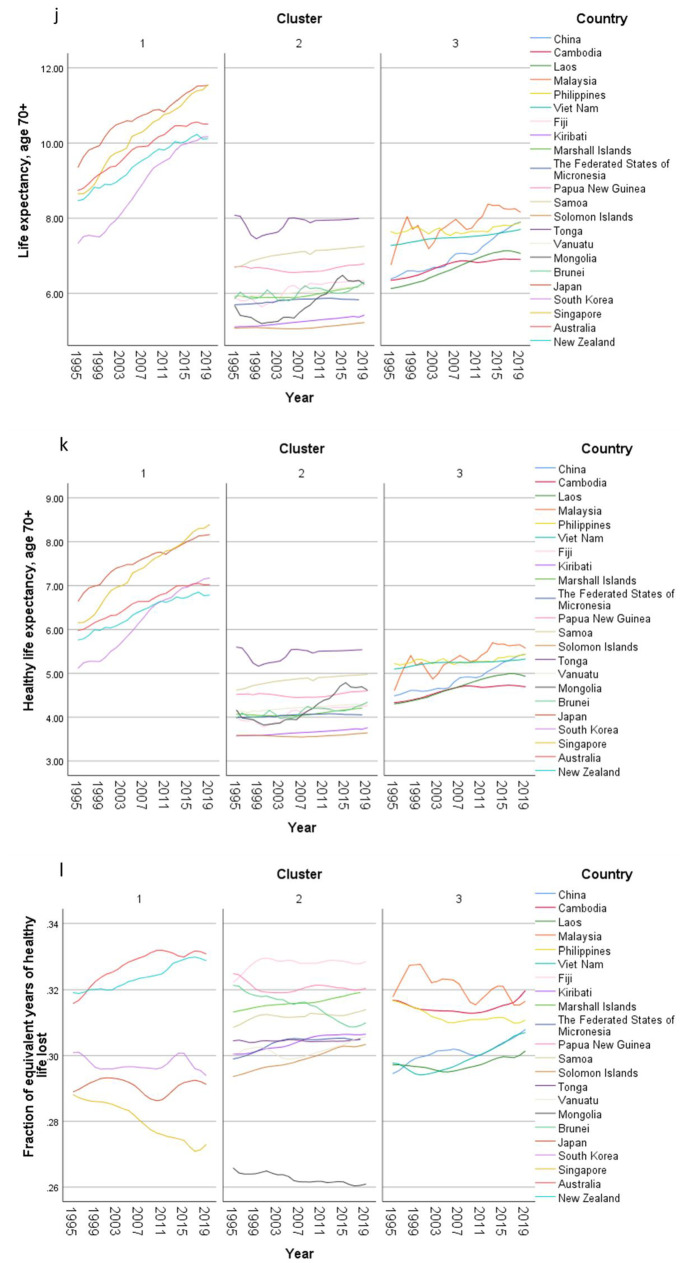
Graphs of sociodemographic and health-related variables over time by clusters, 1995–2019. [*GDP = gross domestic product. PPP = purchasing power parity. THS = total health spending*]

**Fig. 2:**
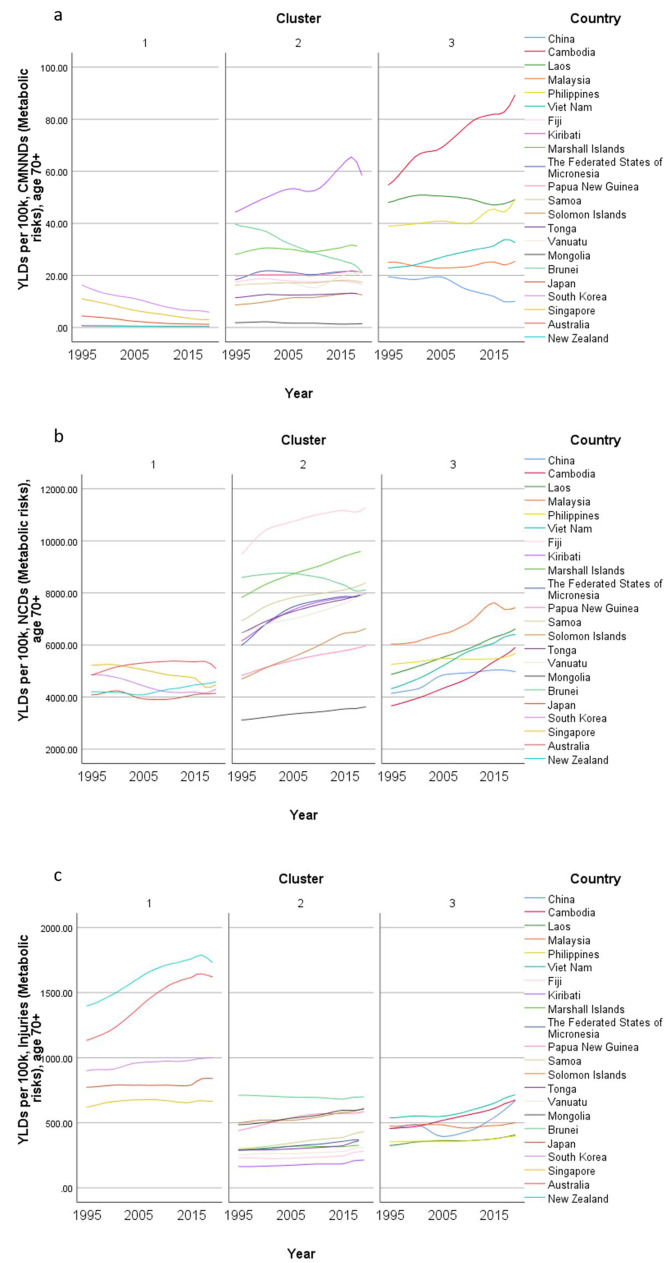

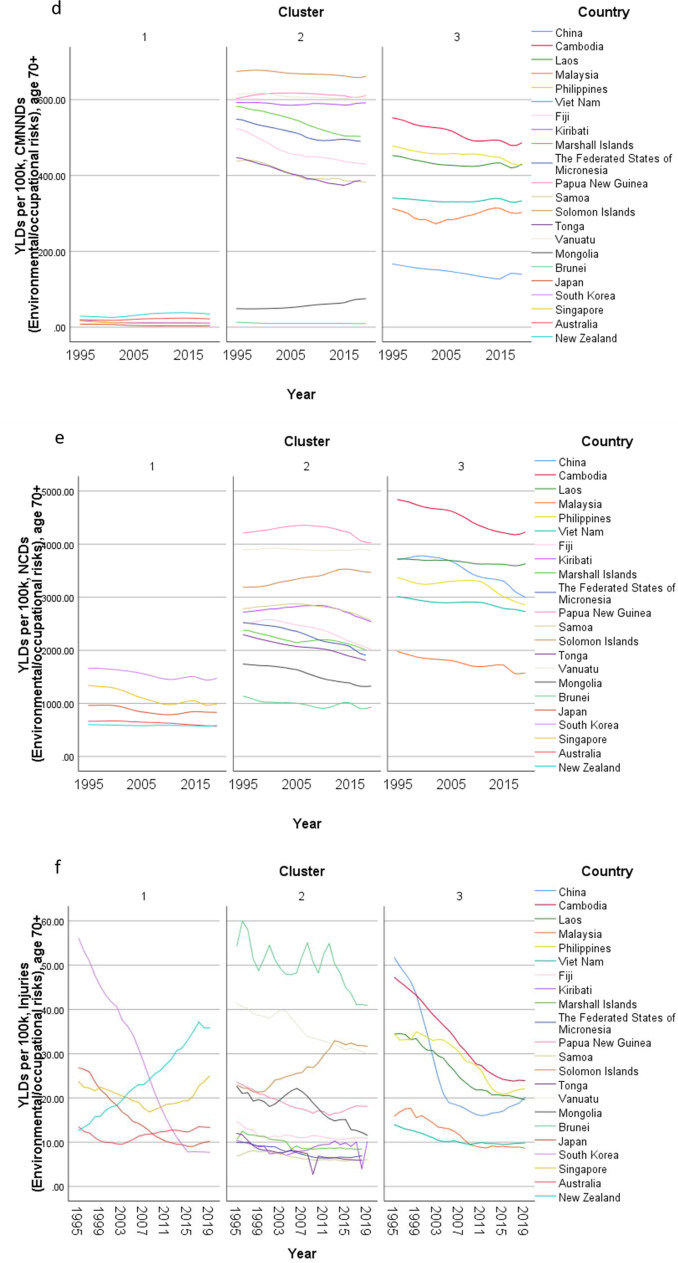

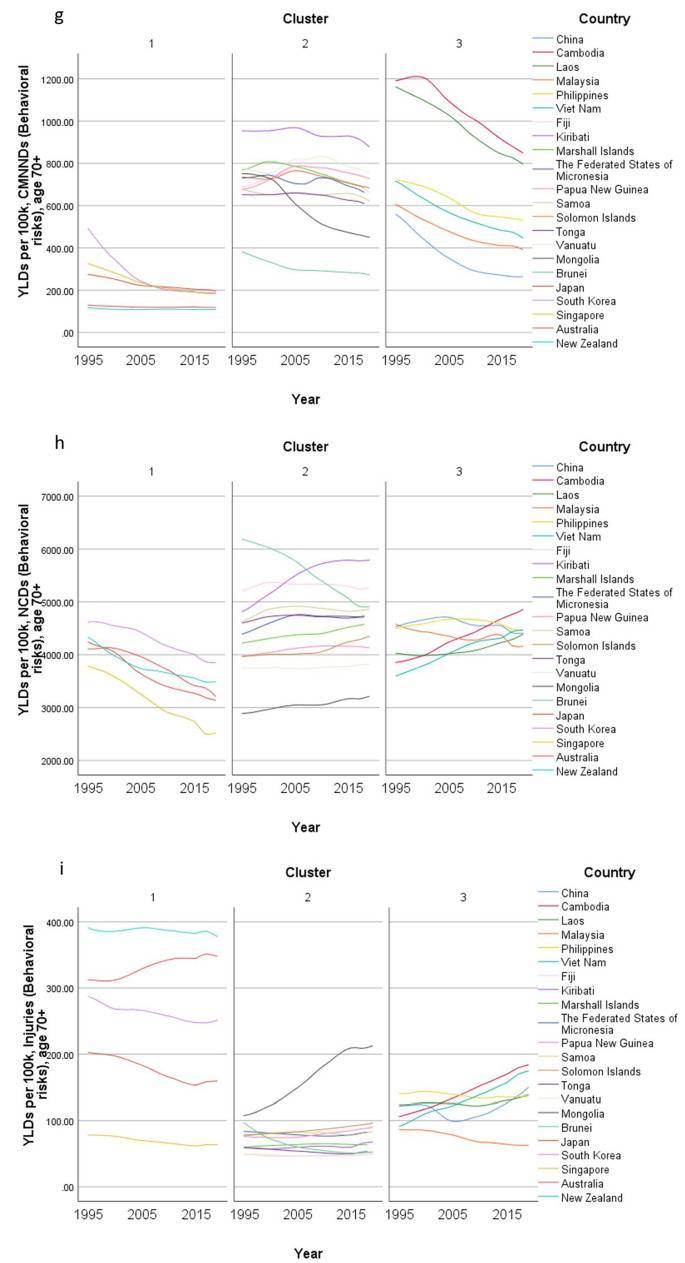
Graphs of YLD rate (per 100 K) risk factors over time by clusters in older adults, 1995–2019. YLD = years lived with disability. [*Metabolic risks = i.e., high fasting plasma glucose; high cholesterol; high blood pressure; high BMI; low bone mineral density; impaired kidney function. Environmental /Occupational risks = i.e., unsafe water; sanitation; handwashing; air pollution; exposure to occupational carcinogens. Behavioural risks = i.e., child & maternal malnutrition; tobacco use; alcohol use; drug use; dietary risks; intimate partner violence; childhood maltreatment; unsafe sex; low physical activity. CMNND = communicable, maternal, neonatal and nutritional diseases. NCD = non-communicable disease*]

**Fig. 3:**
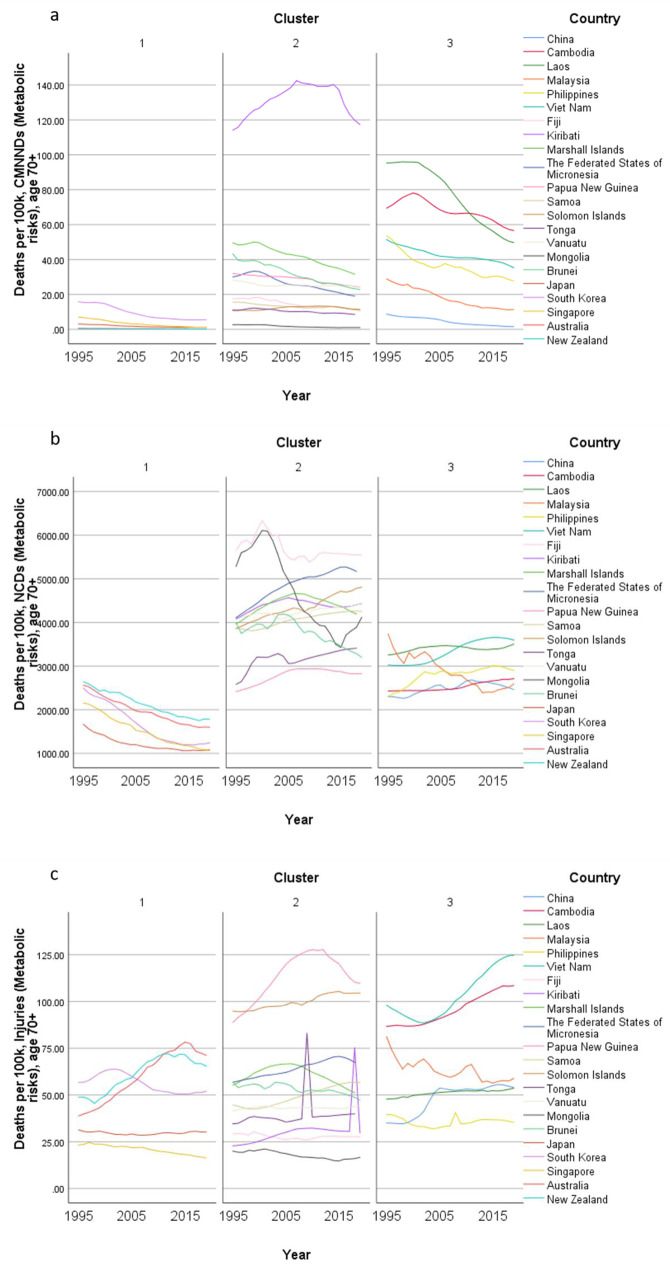

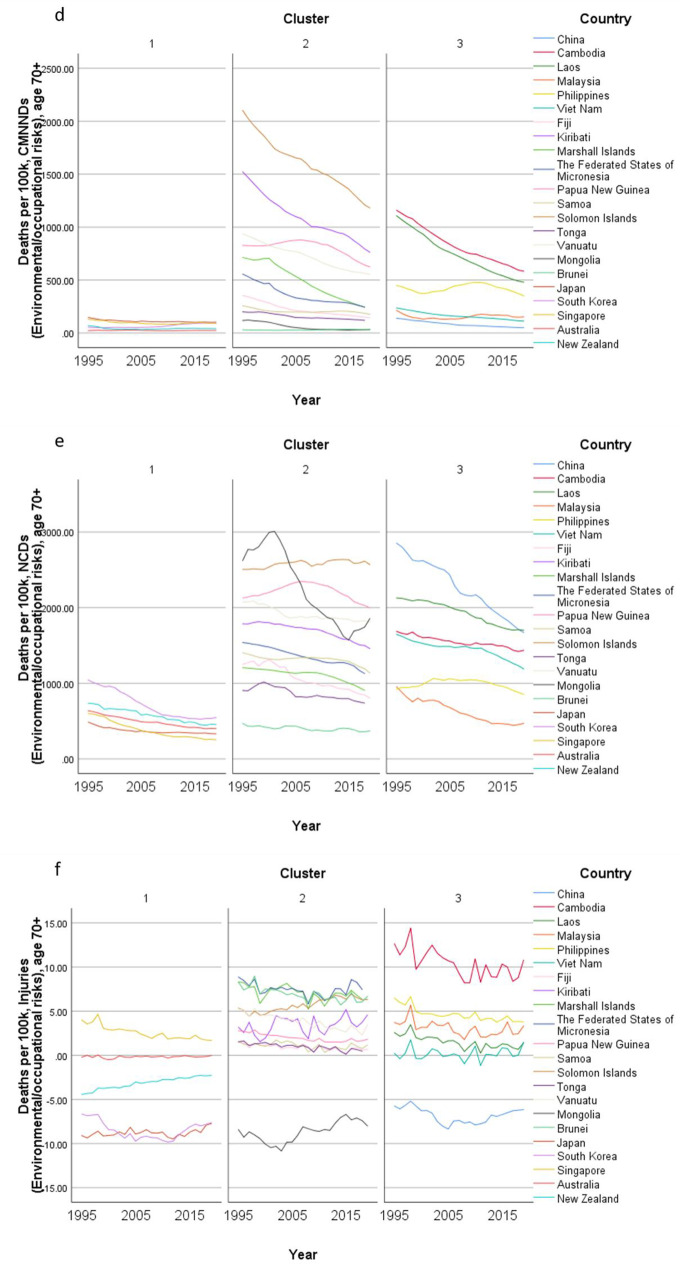

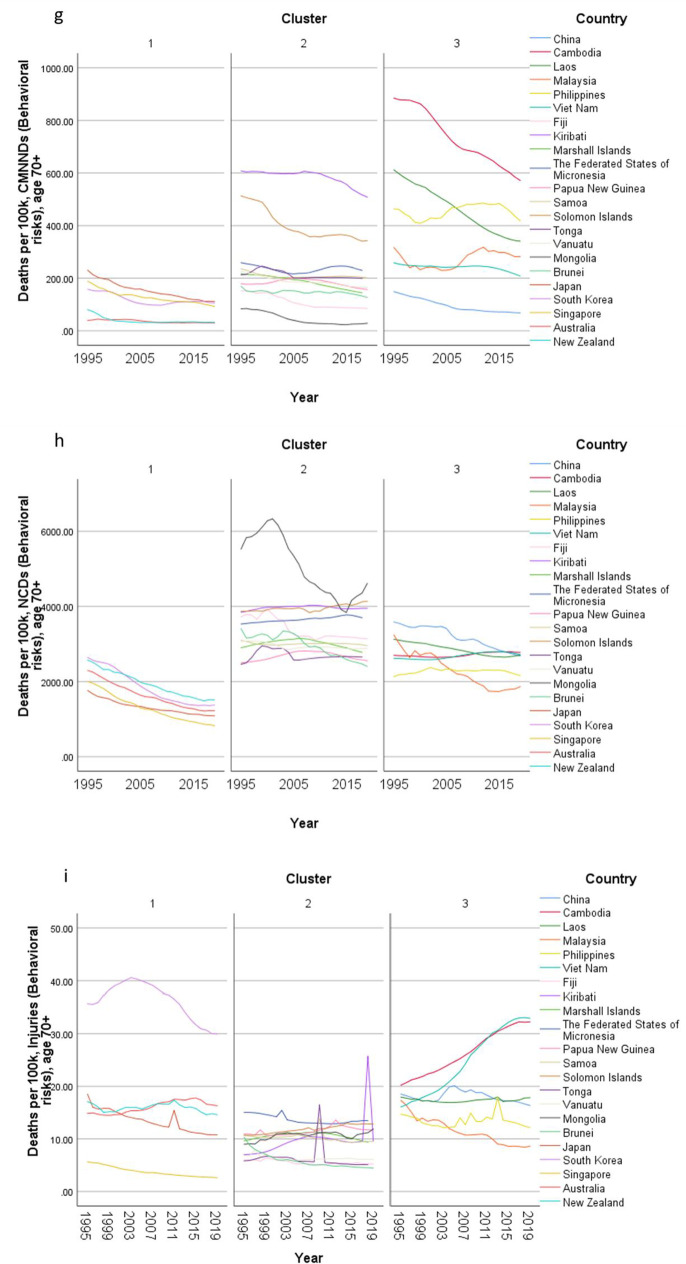
Graphs of mortality rate (per 100 K) over time by clusters, 1995–2019. [*Metabolic risks = i.e., high fasting plasma glucose; high cholesterol; high blood pressure; high BMI; low bone mineral density; impaired kidney function. Environmental /Occupational risks = i.e., unsafe water; sanitation; handwashing; air pollution; exposure to occupational carcinogens. Behavioural risks = i.e. child & maternal malnutrition; tobacco use; alcohol use; drug use; dietary risks; intimate partner violence; childhood maltreatment; unsafe sex; low physical activity. CMNND = communicable, maternal, neonatal and nutritional diseases. NCD = non-communicable disease*]

Concerning country-specific disease burden, a number of developing countries had higher YLD rate and mortality rate of NCDs than other countries. For instance, the YLD rate and mortality rate of NCDs attributable to metabolic risks were higher in Fiji than the other countries. Mongolia had higher mortality rates of NCDs attributable to behavioral risks than other nations. Moreover, the YLD rate of NCDs related to environmental/ occupational risks were higher in Cambodia, Laos, Papua New Guinea, and Vanuatu than the rates in other economies. Country profiles for their demographic and health-related characteristics as well as all risk factors in relation to YLD and mortality in the three clusters are shown in Figs. 1, 2 and 3 respectively. Of note in these figures is that SDI over time has been improving in all countries, the government spending per THS on the Pacific Islands in cluster 2 is extremely volatile as is the THS per GDP, the relatively low THS per capita both in cluster 2 and 3, the increasing life expectancy at age 70 which is more profound in cluster 1 (developed countries) and less so in cluster 2 countries, and HALE at 70 + which show minimal change over time beyond cluster 1 (Fig. 1). Figures 2 and 3 show a clear rise in NCD-related risks in all three clusters but also the different risks across the 22 countries examined.

## Correlations

The SDI, HAQI frontier, GDP per person, education years, fertility rate, and THS per capita had stronger correlations with the YLD rates of (1) Communicable, Maternal, Neonatal, and Nutritional Diseases (CMNNDs) associated with the level 1 risks (aggregate of environmental and occupational risks, behavioral risks, and metabolic risks), (2) NCDs attributable to environmental/ occupational risks, and (3) injuries related to metabolic risks than other factors (Table 3). On the other hand, the correlations between the sociodemographic variables and the mortality rates of CMNNDs and NCDs attributable to the level 1 risk groups were stronger than other correlations (Table 4). The YLD rates of (1) CMNNDs related to level 1 risks, (2) NCDs attributable to behavioral risks or environmental/ occupational risks, as well as (3) injuries associated with metabolic risks or environmental/ occupational risks had stronger correlations with life expectancy, HALE, and LHE at 70 + than other YLD rates (Table 5). In addition, the associations between LHE fraction at 70 + and the CMNNDs attributable to metabolic risks or environmental/ occupational risks were much weaker than other correlations. As for mortality, the rates of CMNNDs, NCDs as well as injuries attributable to environmental/ occupational risks had stronger correlations with LE, HALE, and LHE at 70 + than other rates. In comparison, injuries related to metabolic risks and behavior risks had weaker associations with the life expectancy parameters. It was interesting to see that there were generally low correlations between YLD and mortality with the ratio of health spending to GDP or total health spending, such as THS per GDP, government spending per THS, and private spending per THS. Lower SDI, HAQI, GDP or THS per person were associated with higher death rates of NCD ascribed to metabolic risks as well as behavioral risks.

**Table 3:** Correlations between sociodemographic factors, health spending, and YLD rates (per 100 K) of risk-cause pairs in adults 70 + years old, in the pooled sample

	Met, CMNND	Met, NCD	Met, Inj	Env/Occ, CMNND	Env/Occ, NCD	Env/Occ, Inj	Beh, CMNND	Beh, NCD	Beh, Inj
Sociodemographic index (SDI)	-0·581**	-0·061*	0·503**	-0·815**	-0·866**	-0·197**	-0·718**	-0·110**	0·168**
HAQI frontier	-0·590**	-0·018	0·526**	-0·799**	-0·893**	-0·162**	-0·707**	-0·122**	0·157**
GDP per capita in PPP	-0·495**	-0·127**	0·593**	-0·856**	-0·781**	-0·055	-0·703**	-0·085**	0·243**
Mean years of education, age 25–29	-0·549**	0·028	0·364**	-0·648**	-0·808**	-0·271**	-0·640**	-0·087**	0·140**
Fertility per 1000, age 10–24	0·362**	0·150**	-0·500**	0·808**	0·629**	0·185**	0·622**	0·040	-0·187**
Total health spending per capita in PPP	-0·522**	-0·082**	0·529**	-0·783**	-0·874**	-0·190**	-0·691**	-0·111**	0·201**
Total health spending per GDP	-0·097**	-0·167**	0·092**	-0·040	-0·113**	-0·200**	-0·112**	0·0028	0·230**
Government spending per THS	-0·437**	0·206**	0·077**	-0·117**	-0·424**	-0·093**	-0·276**	0·051	-0·067*
Prepaid private spending per THS	-0·183**	-0·231**	0·244**	-0·440**	-0·288**	-0·053	-0·286**	-0·159**	0·165**
Out-of-pocket spending per THS	0·271**	-0·313**	0·144**	-0·270**	0·071*	0·054	0·035	-0·070*	0·185**

*p < 0·05, **p < 0·01. YLD: years lived with disability. HAQI frontier: healthcare access and quality index computed based on relationship between HAQI and SDI. GDP: gross domestic product. PPP: purchasing power parity. THS: total health spending. Met: metabolic risks (i.e. high fasting plasma glucose; high cholesterol; high blood pressure; high BMI; low bone mineral density; impaired kidney function). Env/Occ: environmental/occupational risks (i.e., unsafe water; sanitation; handwashing; air pollution; exposure to occupational carcinogens). Beh: behavioral risks (i.e., child & maternal malnutrition; tobacco use; alcohol use; drug use; dietary risks; intimate partner violence; childhood maltreatment; unsafe sex; low physical activity). CMNND: communicable, maternal, neonatal and nutritional diseases. NCD: non-communicable disease. Inj: injuries

**Table 4:** Correlations between sociodemographic factors, health spending, and mortality rates (per 100 K) of risk-cause pairs in adults 70 + years old, in the pooled sample

	Met, CMNND	Met, NCD	Met, Inj	Env/Occ, CMNND	Env/Occ, NCD	Env/Occ, Inj	Beh, CMNND	Beh, NCD	Beh, Inj
Sociodemographic index (SDI)	-0·698**	-0·579**	-0·173**	-0·830**	-0·834**	-0·396**	-0·567**	-0·548**	-0·089**
HAQI frontier	-0·696**	-0·501**	-0·146**	-0·871**	-0·835**	-0·325**	-0·609**	-0·508**	-0·113**
GDP per capita in PPP	-0·617**	-0·489**	-0·131**	-0·846**	-0·779**	-0·377**	-0·568**	-0·469**	-0·063*
Mean years of education, age 25–29	-0·650**	-0·497**	-0·184**	-0·729**	-0·796**	-0·350**	-0·520**	-0·519**	-0·088**
Fertility per 1000, age 10–24	0·509**	0·574**	0·024	0·655**	0·606**	0·450**	0·378**	0·441**	-0·023
Total health spending per capita in PPP	-0·643**	-0·551**	-0·126**	-0·796**	-0·799**	-0·334**	-0·534**	-0·506**	-0·057*
Total health spending per GDP	-0·114**	-0·092**	0·104**	0·056*	-0·0013	-0·180**	0·036	-0·040	0·189**
Government spending per THS	-0·349**	0·118**	-0·142**	-0·273**	-0·264**	-0·075**	-0·272**	0·0012	-0·161**
Prepaid private spending per THS	-0·303**	-0·434**	-0·090**	-0·338**	-0·328**	-0·313**	-0·302**	-0·378**	0·036
Out-of-pocket spending per THS	0·102**	-0·404**	0·030	-0·132**	-0·084**	-0·106**	0·055*	-0·214**	0·153**

*p < 0·05, **p < 0·01. HAQI frontier: healthcare access and quality index computed based on relationship between HAQI and SDI. GDP: gross domestic product. PPP: purchasing power parity. THS: total health spending. Met: metabolic risks (i.e., high fasting plasma glucose; high cholesterol; high blood pressure; high BMI; low bone mineral density; impaired kidney function). Env/Occ: environmental/occupational risks (i.e., unsafe water; sanitation; handwashing; air pollution; exposure to occupational carcinogens). Beh: behavioral risks (i.e., child & maternal malnutrition; tobacco use; alcohol use; drug use; dietary risks; intimate partner violence; childhood maltreatment; unsafe sex; low physical activity). CMNND: communicable, maternal, neonatal and nutritional diseases. NCD: non-communicable disease. Inj: injuries

**Table 5:** Correlations between YLD rates and mortality rates (per 100 K) of risk-cause pairs and the life expectancy and LHE fraction at 70 + years old, in the pooled sample

	LE at 70 +	HALE at 70 +	LHE at 70 +	LHE fraction at 70 +
YLD (Met, CMNND)	-0·494**	-0·492**	-0·478**	-0·071**
YLD (Met, NCD)	-0·235**	-0·300**	-0·097**	0·470**
YLD (Met, Inj)	0·528**	0·494**	0·562**	0·288**
YLD (Env/Occ, CMNND)	-0·644**	-0·675**	-0·565**	0·109**
YLD (Env/Occ, NCD)	-0·455**	-0·434**	-0·472**	-0·178**
YLD (Env/Occ, Inj)	-0·340**	-0·275**	-0·452**	-0·553**
YLD (Beh, CMNND)	-0·377**	-0·422**	-0·270**	0·315**
YLD (Beh, NCD)	-0·448**	-0·417**	-0·487**	-0·246**
YLD (Beh, Inj)	0·028	0·080**	-0·077**	-0·369**
Mortality (Met, CMNND)	-0·619**	-0·609**	-0·612**	-0·159**
Mortality (Met, NCD)	-0·788**	-0·812**	-0·699**	0·099**
Mortality (Met, Inj)	-0·064*	-0·123**	0·046	0·456**
Mortality (Env/Occ, CMNND)	-0·552**	-0·572**	-0·501**	-0·0055
Mortality (Env/Occ, NCD)	-0·615**	-0·569**	-0·671**	-0·374**
Mortality (Env/Occ, Inj)	-0·518**	-0·524**	-0·491**	-0·063*
Mortality (Beh, CMNND)	-0·549**	-0·524**	-0·585**	-0·308**
Mortality (Beh, NCD)	-0·877**	-0·828**	-0·921**	-0·421**
Mortality (Beh, Inj)	-0·245**	-0·184**	-0·355**	-0·491**

*p < 0·05, **p < 0·01. YLD: years lived with disability. LE: life expectancy. HALE: healthy life expectancy. LHE: equivalent years of healthy life lost. Met: metabolic risks (i.e., high fasting plasma glucose; high cholesterol; high blood pressure; high BMI; low bone mineral density; impaired kidney function). Env/Occ: environmental/occupational risks (i.e., unsafe water; sanitation; handwashing; air pollution; exposure to occupational carcinogens). Beh: behavioral risks (i.e., child & maternal malnutrition; tobacco use; alcohol use; drug use; dietary risks; intimate partner violence; childhood maltreatment; unsafe sex; low physical activity). CMNND: communicable, maternal, neonatal and nutritional diseases. NCD: non-communicable disease. Inj: injuries

## Correlation matrix

**Fig. 4:**
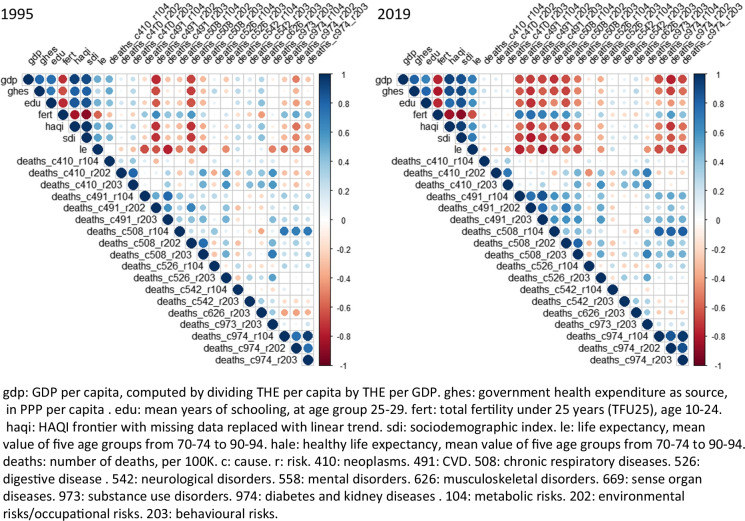
Correlation matrix of sociodemographic variables, life expectancy, and death rates in 1995 and 2019

In relation to specific disease groups, among all correlations with life expectancy or HALE, only the correlation between HALE and YLDs attributable to neurological disorder and behavioral risks in 1995 were non-significant (rho = 0.01, *p* = 0.25). All other correlations were significant (*p* < 0.05). As shown in Fig. 4, the correlations between life expectancy and deaths from CVD, CRD, DM and renal diseases were strong, and their correlations with SDI became stronger in 2019 compared with 1995.

**Fig. 5:**
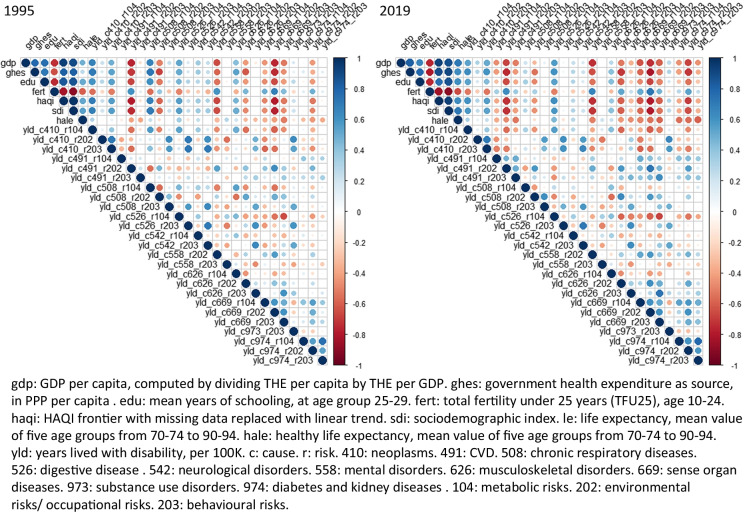
Correlation matrix of sociodemographic variables, HALE, and YLDs rates in 1995 and 2019

Concerning YLDs, in 1995, HALE showed moderate to weak correlations with YLDs (Fig. 5). However, in 2019, the correlations between HALE and YLDs from CVD, musculoskeletal disorders, sense organ diseases, and diabetes and renal diseases became stronger. Noticeably, the correlations between HALE and digestive diseases due to metabolic risk, as well as mental health disorders due to environmental /occupational risks, also became stronger. Overall, the correlation between SDI and CVD and sense organ diseases became stronger, although the correlation between SDI and CRD became weaker. The correlation between SDI and cancer due to metabolic risk and behavioral risk, as well as digestive diseases due to metabolic risk, were stronger over time. Moreover, the correlation between SDI and musculoskeletal disorders, DM and renal disease, and mental health disorders due to environmental/ occupational risk became stronger in 2019 too.

## Regression analyses

In the linear mixed effects regression analyses, SDI was the most significant independent variable of all-risk and all-cause YLD rate and mortality rate (Tables 6, 7). Higher SDI was associated with around 1600 years decrease in YLDs per 100K of population and a drop in nearly 1500 deaths per 100K of population, when health spending was adjusted for (Model I). When HAQI was controlled for in the models (Model II), health spending was associated with almost 240 less YLDs per 100K and around 150 less deaths per 100K for those at 70+ years old. The mortality rate in females was significantly lower than males by around 1200 deaths per 100K of population. One unit of THS per capita was associated with a fall in more than 150 deaths per 100K of population (model II).

**Table 6:** Linear mixed effect models to assess YLD rates (per 100 K) with respect to sociodemographic factors and health spending

	Estimate	95% CI	t (df)	F (edf)
Intercept	11,560·1	[10190·82, 12,929·37]	16·55 (1294)***	
Sex	-528·35	[-1394·26, 337·57]	-1·2 (1294)	
Year	386·09	[311·11, 461·06]	10·09 (1294)***	25·98 (7·47)***
SDI	-1514·41	[-1934·03, -1094·79]	-7·07 (1294)***	30·48 (8·36)***
HAQI frontier	-105·15	[-251·89, 41·6]	-1·4 (1294)	1·34 (3·63)
THS per capita	11·15	[-45·45, 67·74]	0·39 (1294)	0·15 (1)

Adjusted *R* squared = 0·26. *p < 0·05, **p < 0·01, ***p < 0·001. YLD: years lived with disability. SDI: socio-demographic index. HAQI frontier: healthcare access and quality index computed based on relationship between HAQI and SDI. THS: total health spending

**Table 7:** Linear mixed effect models to assess mortality rates (per 100 K) with respect to sociodemographic factors and health spending

	Estimate	95% CI	t (df)	F (edf)
Intercept	7105·22	[6256·52, 7953·91]	16·41 (1294)***	
Sex	-1245·99	[-1782·11, -709·86]	-4·56 (1294)***	
Year	146·1	[17·78, 274·42]	2·23 (1294)*	3·57 (5·69)**
SDI	-1498·92	[-1944·8, -1053·03]	-6·59 (1294)***	32·06 (2·35)***
HAQI frontier	49·12	[-371·06, 469·29]	0·23 (1294)	2·78 (6·5)**
THS per capita	-83·51	[-204·11, 37·09]	-1·36 (1294)	1·91 (1·07)

Adjusted *R* squared = 0·71. *p < 0·05, **p < 0·01, ***p < 0·001. SDI: socio-demographic index. HAQI frontier: healthcare access and quality index computed based on relationship between HAQI and SDI. THS: total health spending

When generalised additive mixture modelling was applied, it was shown that between 1995 and 2019, the change in LHE at 70 + was minimal (Table 8). Overall, YLD rates of CMNNDs attributable to level 1 (metabolic) risks were associated with a lower LHE when other YLD rates were adjusted for. For example, one YLD per 100 K of population related to behavioral risk was associated with a decrease in LHE by 0.2 years. One YLD per 100 K of population due to metabolic risk was correlated with a 4-month less of LHE. However, YLD rates of NCDs and injuries attributable to metabolic risks were associated with a rise in LHE. One YLD per 100 K of population related to NCD or injuries was associated with 0.25 or 0.2 years more in LHE. YLD rate of NCD associated with behavioral risks was correlated with a fall in LHE, but the result was only significant in the non-linear relationship. YLD rate of injuries attributable to environmental/occupational risks was associated with longer LHE.

**Table 8:** Generalized additive mixture models to assess the LHE with respect to YLD rates (per 100 K) of risk-cause pairs, for both sexes

	Estimate	95% CI	t (df)	F (edf)
Intercept	2·17	[2·04, 2·31]	31·39 (1586)***	
YLD (Met, CMNND)	-0·32	[-0·52, -0·11]	-3·03 (1586)**	4·22 (6·28)***
YLD (Met, NCD)	0·25	[0·15, 0·36]	4·62 (1586)***	13·67 (1·92)***
YLD (Met, Inj)	0·2	[0·12, 0·29]	4·91 (1586)***	24·13 (1)***
YLD (Env/Occ, CMNND)	0·02	[-0·08, 0·13]	0·47 (1586)	0·22 (1)
YLD (Env/Occ, NCD)	0·01	[-0·08, 0·09]	0·16 (1586)	0·02 (1)
YLD (Env/Occ, Inj)	0·77	[0·69, 0·86]	17·77 (1586)***	74·64 (8·88)***
YLD (Beh, CMNND)	-0·18	[-0·34, -0·03]	-2·28 (1586)*	3·93 (3·35)**
YLD (Beh, NCD)	-0·11	[-0·37, 0·14]	-0·87 (1586)	6·33 (6·8)***
YLD (Beh, Inj)	-0·08	[-0·22, 0·05]	-1·24 (1586)	1·62 (2·44)

Adjusted *R* squared = 0·47. *p < 0·05, **p < 0·01, ***p < 0·001. Model was adjusted for years and clusters. LHE: equivalent lost healthy years. YLD: years lived with disability. Met: metabolic risks (i.e., high fasting plasma glucose; high cholesterol; high blood pressure; high BMI; low bone mineral density; impaired kidney function). Env/Occ: environmental/occupational risks (i.e., unsafe water; sanitation; handwashing; air pollution; exposure to occupational carcinogens). Beh: behavioral risks (i.e., child & maternal malnutrition; tobacco use; alcohol use; drug use; dietary risks; intimate partner violence; childhood maltreatment; unsafe sex; low physical activity). CMNND: communicable, maternal, neonatal and nutritional diseases. NCD: non-communicable disease. Inj: injuries

The change in life expectancy was non-significant when mortality rates were controlled for between 1995 and 2019 (Table 9). Overall, the death rates of NCDs were associated with lower life expectancy. The mortality rates of NCDs with respect to behavioral risks and metabolic risks were associated with 1·9 and 0·4 years decrease in life expectancy respectively. The mortality of CMNNDs attributable to environmental/ occupational risks and behavioral risks were associated with around 0·8 and 0·1 years decrease in life expectancy respectively. In addition, the death rate of injuries attributable to metabolic risks and behavioral risks were associated with shorter life expectancy when other risk related mortality rates were adjusted for.

**Table 9:** Generalized additive mixture models to assess life expectancy with respect to mortality rates (per 100 K) of risk-cause pairs, for both sexes

	Estimate	95% CI	t (df)	F (edf)
Intercept	7·24	[7·03, 7·45]	67·67 (1586)***	
Mortality (Met, CMNND)	-0·03	[-0·13, 0·06]	-0·69 (1586)	0·3 (1·26)
Mortality (Met, NCD)	-0·42	[-0·67, -0·17]	-3·26 (1586)**	14·45 (7·06)***
Mortality (Met, Inj)	-0·36	[-1·24, 0·53]	-0·79 (1586)	38·11 (7·08)***
Mortality (Env/Occ, CMNND)	-0·82	[-1·1, -0·53]	-5·61 (1586)***	6·85 (7·95)***
Mortality (Env/Occ, NCD)	0·17	[-0·15, 0·49]	1·06 (1586)	1·84 (6·65)
Mortality (Env/Occ, Inj)	-0·003	[-0·03, 0·02]	-0·25 (1586)	0·06 (1)
Mortality (Beh, CMNND)	-0·13	[-0·24, -0·02]	-2·31 (1586)*	5·34 (1)*
Mortality (Beh, NCD)	-1·88	[-2·3, -1·45]	-8·66 (1586)***	18·2 (8·75)***
Mortality (Beh, Inj)	-1·62	[-2·18, -1·06]	-5·66 (1586)***	94·4 (8·85)***

Adjusted *R* squared = 0·84. *p < 0·05, **p < 0·01, ***p < 0·001. Model was adjusted for years and clusters. Met: metabolic risks (i.e., high fasting plasma glucose; high cholesterol; high blood pressure; high BMI; low bone mineral density; impaired kidney function). Env/Occ: environmental/occupational risks (i.e., unsafe water; sanitation; handwashing; air pollution; exposure to occupational carcinogens). Beh: behavioral risks (i.e., child & maternal malnutrition; tobacco use; alcohol use; drug use; dietary risks; intimate partner violence; childhood maltreatment; unsafe sex; low physical activity). CMNND: communicable, maternal, neonatal and nutritional diseases. NCD: non-communicable disease. Inj: injuries

## Discussion

A key finding in this study on older adults over the age of 70 years in the Western Pacific Region is the categorisation of 22 countries into three clusters, which can have implications for regional policy planning, for example by the World Health Organization. Life expectancy and HALE at 70 + increased steadily over the years, which is more evident in developed countries and also with some upward trends in developing countries. It creates an urgency for health planning to focus on factors affecting health outcomes in older adults across the region. Other important messages from this study include that SDI is the most significant factor to both YLD rate and mortality rate. Health system indicators such as health spending was not consistently linked with disease burden on older adults when SDI and HAQI were taken into account. The results suggested that the overall sociodemographic development could be an important determinant of health outcomes beyond health spending and healthcare access and quality. Hence, integrated health planning strategies are needed. YLDs and mortality from NCDs, while being key issues in all three clusters, increased considerably in the developing countries. Wide variations in health risks were also observed in the Western Pacific countries, but no major change was, however, shown from 1995 to 2019 for many of such risks. In addition, women had better YLDs rates and mortality outcomes than men.

The findings in the current study in part are consistent with the results from the previous GBD studies. Additionally, the results provided insights into the associations between sociodemographic factors, disease burden, and life expectancy as well as the proportion of years spent in poor health among older adults in the Western Pacific countries, which has not been done before. This study found that though developed countries (cluster 1) had higher total health spending (THS) per capita and lower YLD rate and mortality rate than developing countries (cluster 2 and 3), THS per person was not the most significant factor to disease burden when SDI was adjusted for. Also, the gap in YLDs rate between developing countries and developed countries has been growing wider along with healthy life expectancy. The developing countries should particularly be the targets of assistance in international public health interventions.

The sociodemographic index was found to be a more important factor to YLD rate and mortality rate than THS per capita in the current study. As high SDI reflects higher GDP and education years and lower fertility rates, it may be a relatively distal proxy compared with other factors such as the availability and accessibility [31], quality [32] and error in diagnostic tests and treatments [33] which could have differential impact on the YLD and mortality between countries. Further global study of country-specific estimates with respect to proximal, focused, and modifiable factors to disease burden is required. There is limited work in interventions to improve socioeconomic status as a driver to health outcomes in the ageing population. However, work in other age groups or conditions suggest that structural interventions (see examples in: https://www.who.int/social_determinants/knowledge_networks/phconditions/structural_interventions.pdf) may improve health inequalities and outcomes [34–36].

The risks attributable to NCDs were the major contributors to the disease burden for those aged 70 years or above in the Western Pacific Region by 2019. For example, the YLD rates of diabetes related to hyperglycaemia, ischemic stroke attributable to hypertension, and COPD associated with smoking were the highest among all risk-cause pairs in the literature [37]. The mortality rates of ischemic heart disease associated with hypertension and hypercholesterolemia, as well as cancers related to smoking, were the top-ranked rates in older adults [37].

In terms of country risk profiles, a number of factors are proposed to be associated with an increased risk of NCDs in these countries. With respect to NCDs attributable to metabolic and behavioral risks, the prevalence rate of diabetes as well as obesity and hypertension were found rising among Fijians, and risk factors of CVDs related to diet, exercise, and smoking had compromised the life expectancy which was similar to the epidemiological transition in developed countries earlier [38]. In the Mongolian population, ischaemic heart disease was found to be associated with advanced age, low intake of fruits or vegetables, and substance use [39]. Concerning NCDs attributable to environmental risks, worse lung function and respiratory disorders were found to be associated with indoor air pollutants from burning biomass fuel for cooking, home dust, animal contact, and smoking in rural areas in Laos [40–42]. In the Philippines, a study found that around one fifth of a sample were identified as having COPD, and those who had history of tuberculosis, smoking, or used wood fire for cooking had higher odds of the disease [43].

The findings in the literature and the current study implied that the disease burden in terms of YLD rate and mortality rate of NCDs such as CVDs, diabetes, and chronic lung disease attributable to metabolic risks such as hypertension, hyperglycaemia, and obesity; behavioral risks such as dietary risks and substance use; as well as environmental risks such as household air pollution were contributing to longer LHE and shorter life expectancy at 70 + years particularly in high-risk countries. However, the country cluster was not a significant independent variable of either LHE or life expectancy when time and either YLD rate or mortality rate were statistically controlled for (data not shown in text). Furthermore, the number of years lived in poor health has been growing over the years [1]. Thus, the control of NCDs and their risks should have a high priority in lowering the LHE fraction at older ages.

A final comment is directed towards government health spending and THS per GDP in Pacific Island countries, which were inconsistent over the years, with total health spending at the same time being at a low level. The governments of these countries need to rethink their health spending in a more consistent way and direct more health spending on the areas of higher priority as shown in the results of this analysis, including health promotion programmes in older people related to NCDs. This may include, among other things, specific strategies for a more concrete integration of traditional medicine, which is the main primary health care in many of the communities on Pacific Islands [44, 45].

The findings of our work have a dual set of policy implications. First, it will help Western Pacific countries with a high percentage of aged residents plan, develop, and implement health intervention programmes in order to address and reduce the burden of disability and mortality among older adults. Second, it will help other specific Western Pacific regions, such as low- and middle-income ones, better prepare their preventive strategies and public health systems for future health needs due to the rapid demographic changes.

## Limitations

All limitations of the GBD 2019 publications are also applicable to this study [7, 9, 10], mostly the challenges of capturing sources of uncertainty, lags in data availability, variation in coding practices and other biases, and limitations of existing analytical tools, which may not fully capture temporal analysed trends among the Western Pacific Region countries. Available data sources for the GBD population included, among others surveys, censuses adjusted and unadjusted, and WHO vital registration systems. Additional limitations are that the data analysis could not adjust for other factors influencing LE, HALE, population mortality and YLDs, as for example genetic factors, adherence to specific medication) and changes in the treatment that could also alter the presented estimations. Also, accumulated comorbidities is a common phenomenon in older adults [46], however the nature of GBD data does not allow to account for accumulated comorbidities, an issue that may further limit the true magnitude of our findings. GAM models were tested also without taking into account clusters and equivalent results were confirmed (data not shown in text). As this is a descriptive study, while we analyse a number of correlates and associations among the variables of interest, our analyses cannot conclude causal relationships. Finally, the applied modelling methodologies in this study may not capture fully the trends and patterns of disease burden among older adults living in WPRs.

## Conclusion

The sociodemographic and socioeconomic development was found a more important factor to the disease burden in terms of YLD rate and mortality rate among older adults than the health spending in the Western Pacific Region. However, proximal predictors of YLD rate and mortality rate need to be identified with country-specific parameters estimated to outline a global picture with respect to levels of modifiable risk factors. YLD and mortality of NCDs attributable to certain risks were identified as significant contributors to longer LHE and shorter life expectancy respectively in the context of rising YLD rate. Sub-regional health planning and interventions should be developed and prioritized based on cluster-specific characteristics and disease burden to lower LHE fraction and probably lengthen life expectancy and HALE at 70 + in the Western Pacific Region. Evidence-based work on successful ageing is completely missing from the Pacific Islands [47] as well as much of the developing countries in this region. Leaders of LMICs need to have vision and plan to promote heath knowledge and awareness at personal and governmental levels which is supported with social advancements. Hence, governments and policy makers in the region need to urgently prioritise "healthy ageing" as has been done in other developed countries globally, as both life expectancy is steadily increasing and the burden of NCDs alongside other health risk factors is also growing.

## Supplementary Information

Below is the link to the electronic supplementary material.

Supplementary file1 (DOCX 92 KB)

Supplementary file2 (DOCX 17 KB)

## Data Availability

To download the data used in these analyses, please visit the Global Health Data Exchange GBD 2020 website.

## Code availability

N/A.
