# Supplementary material for: Associations between sociodemographic factors, health spending, disease burden, and life expectancy of older adults (70 + years old) in 22 countries in the Western Pacific Region, 1995–2019: estimates from the Global Burden of Disease (GBD) Study 2019
Source: GeroScience. 2022 Jan 8;44(2):925–51. doi: 10.1007/s11357-021-00494-z (PMC9135952; doi:10.1007/s11357-021-00494-z)
Supplement: Supplementary file 1 — Supplementary file1 (DOCX 92 KB) [file 11357_2021_494_MOESM1_ESM.docx]

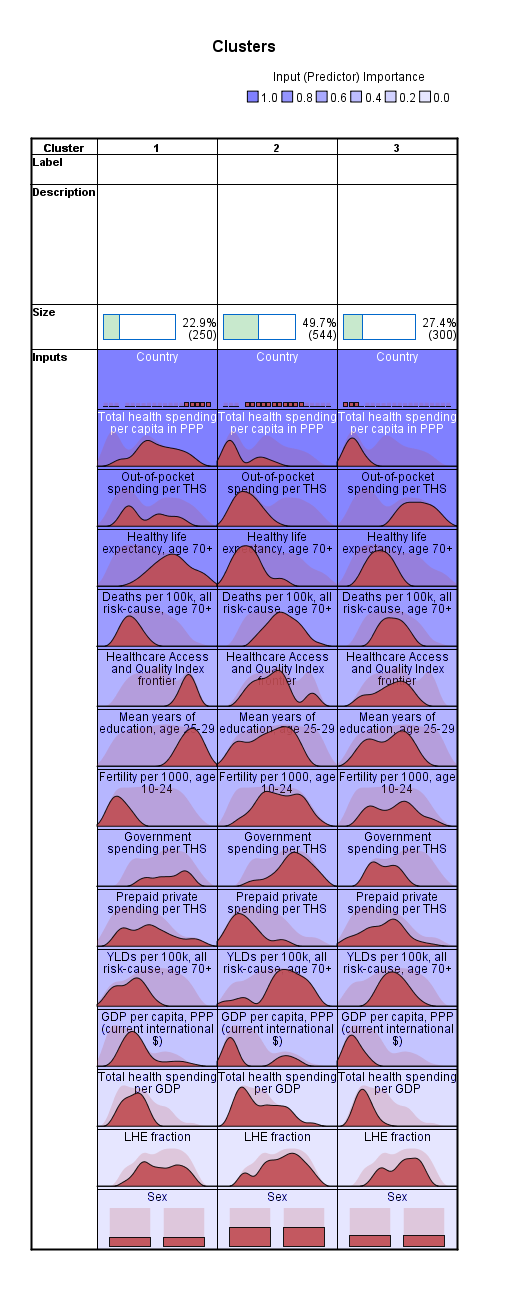


**Supplementary Figure 1: Absolute distributions of frequencies of input variables and their levels of importance**

THS = total health spending. GDP = gross domestic product. PPP = purchasing power parity. YLD = years lived with disability. LHE = equivalent lost healthy yea
