# Supplementary material for: Associations between sociodemographic factors, health spending, disease burden, and life expectancy of older adults (70 + years old) in 22 countries in the Western Pacific Region, 1995–2019: estimates from the Global Burden of Disease (GBD) Study 2019
Source: GeroScience. 2022 Jan 8;44(2):925–51. doi: 10.1007/s11357-021-00494-z (PMC9135952; doi:10.1007/s11357-021-00494-z)
Supplement: Supplementary file 2 — Supplementary file2 (DOCX 17 KB) [file 11357_2021_494_MOESM2_ESM.docx]

**Supplementary appendix 1**

Two-step cluster analysis was used to explore the number and characteristics of clusters in the data with respect to Western Pacific Region. The categorical variables were country and sex. The continuous variables were GDP per capita, education years, fertility rate, THS per capita, THS per GDP, government spending per THS, prepaid private spending per THS, OOP spending per THS, HAQI frontier, YLD rate, mortality rate, HALE, and LHE fraction at 70+ years old. The pre-cluster step used a sequential clustering approach ^1^. It scanned the records one by one and decided if the current record should merge with the previously formed clusters or start a new cluster based on the distance criterion. The similarity between clusters were evaluated with log-likelihood distances. Then we implement the procedure by constructing a modified cluster feature (CF) tree ^2^. In the CF tree tuning, the threshold of initial distance change was set at zero. The maximum number of tree levels, branches per leaf node, and possible nodes were set at three, eight, and 585 respectively. The number of clusters was selected based on the ratio of BIC change and the ratio of distance measure. In the outputs, the cluster quality was evaluated with the silhouette measure of cohesion and separation. In addition, the importance of each input (variable) was quantified as a score ranging between 0 (the least important) and 1 (the most important).

Spearman’s rank-order correlation tests were run to examine the associations between the sociodemographic factors as well as health spending and the YLD rate and mortality rate of risk-cause pairs. Moreover, correlation tests were conducted to investigate the associations between the YLD rate and mortality rate of risk-cause pairs and the LHE fraction and life expectancy as well as LHE and HALE at age 70-94 years. YLD rates and mortality rates in the correlation tests with LHE and LE at 70+ respectively were further analysed in regression models. The significance level was set at 0·05.

Linear mixed effects (LME) models and generalized additive mixture (GAM) models were built to estimate the parameters of YLD rates and mortality rates with respect to sex, year, SDI, HAQI frontier, and THS per capita. Furthermore, LHE at 70+ was regressed on clusters, year, and YLD rates of risk-cause pairs. On the other hand, life expectancy was the dependent variable of the cluster, year, and mortality rates of the risk-cause pairs. The parameter estimation method was restricted maximum likelihood, and the correlation structure across years was specified as first-order autoregressive. The smoothing bases for continuous independent variables were penalized thin plate splines ^3^. The basis dimension (k) of each smooth term was set at ten ^4^. Residuals were checked and there were no obvious violations of the assumptions of normality and homoscedasticity. The software used for correlation tests and cluster analysis was IBM SPSS 25 (Armonk, NY: IBM Corp.) and R was used to run LME and GAM models ^3^.

References

1. Theodoridis S, Koutroumbas K. Pattern recognition. New York: Academic Press; 1999.

2. Zhang T, Ramakrishnon R, Livny M. BIRCH: An efficient data clustering method for very large databases. ACM SIGMOD Conference on Management of Data; 1996; Montreal, Canada; 1996. p. 103–14.

3. Wood S. Mixed GAM computation vehicle with automatic smoothness estimation. 2019. https://cran.r-project.org/web/packages/mgcv/mgcv.pdf (accessed 2nd Aug 2020).

4. Wood SN. Generalized additive models: An introduction with R. 2 ed: Chapman and Hall/CRC Press.; 2017.
